# Extra-Neutralizing FcR-Mediated Antibody Functions for a Universal Influenza Vaccine

**DOI:** 10.3389/fimmu.2019.00440

**Published:** 2019-03-18

**Authors:** Carolyn M. Boudreau, Galit Alter

**Affiliations:** ^1^Ragon Institute of MGH, MIT, and Harvard, Cambridge, MA, United States; ^2^Harvard Ph.D. Program in Virology, Division of Medical Sciences, Harvard University, Boston, MA, United States

**Keywords:** influenza, antibody, Fc receptor, vaccine, ADCC, glycosylation, adjuvant

## Abstract

While neutralizing antibody titers measured by hemagglutination inhibition have been proposed as a correlate of protection following influenza vaccination, neutralization alone is a modest predictor of protection against seasonal influenza. Instead, emerging data point to a critical role for additional extra-neutralizing functions of antibodies in protection from infection. Specifically, beyond binding and neutralization, antibodies mediate a variety of additional immune functions via their ability to recruit and deploy innate immune effector function. Along these lines, antibody-dependent cellular cytotoxicity, antibody-mediated macrophage phagocytosis and activation, antibody-driven neutrophil activation, antibody-dependent complement deposition, and non-classical Fc-receptor antibody trafficking have all been implicated in protection from influenza infection. However, the precise mechanism(s) by which the immune system actively tunes antibody functionality to drive protective immunity has been poorly characterized. Here we review the data related to Fc-effector functional protection from influenza and discuss prospects to leverage this humoral immune activity for the development of a universal influenza vaccine.

## Introduction

Influenza viruses are enveloped negative-strand RNA viruses with segmented genomes that can infect a variety of birds and mammals, including humans (1). Seasonal influenza affects 10–20% of the world's population per year (2), which is estimated to cost $4.6 billion yearly for hospitalizations, doctor's visits, and medications in the United States alone (3). Additionally, influenza causes U.S. employees to miss approximately 17 million workdays due to flu, at an estimated cost of $7 billion a year in sick days and lost productivity (3). Increased infection and mortality occurred during four pandemics in the 20th and 21st centuries, in 1918, 1957, 1968, and 2009 (4), and could occur again if a new strain, such as avian influenzas H5N1 or H7N9, begins to circulate in the human population.

To address this looming threat, the National Institute of Allergy and Infectious Diseases (NIAID) has named the development of a universal influenza vaccine, defined as one that provides protection against symptomatic disease from ≥75% of influenza A strains, as one of its research priorities (5). A key component of the strategic plan is the initial characterization of the correlates of immunity against influenza infection and disease (6). While neutralization has been widely considered the major protective correlate of immunity, hemagglutinination-inhibiting neutralizing antibodies alone have been only modestly linked to protection from seasonal influenza infection, suggesting the involvement of extra-neutralizing antibody functions (7–10). Moreover, currently licensed seasonal influenza vaccines provide only moderate (10–60%) protection against specific strains of seasonal influenza, and little to no protection from emerging pandemic influenza (11). This low efficacy is caused by subtype and strain variability of the two major viral antigens, hemagglutinin (HA) and neuraminidase (NA), as well as by antigenic drift (12). All the available seasonal influenza vaccines, including the inactivated influenza vaccine (IIV), adjuvanted IIV (FluAd), and live attenuated influenza vaccine (LIAV), are given yearly due to limited response durability and the need to induce *de novo* immunity to novel circulating strains. The development of yearly influenza vaccines relies on predictions published by the World Health Organization to determine the strain composition for a given year (13). Due to the long lead times in producing adequate quantities of the vaccine, the strains must be selected roughly 6 months in advance of vaccine administration (13), leading to population vulnerability should a new strain enter circulation. When these predictions did not match seasonal circulating strains, the effectiveness was very low (11).

Beyond efforts to match sequences to ensure seasonal immunity, the current surrogate of protection used to evaluate influenza vaccines is the hemagglutination inhibition (HAI) assay (14). HAI was identified as a predictor of protection from infection in the initial study of egg-grown inactivated vaccine efficacy conducted in 1943 by Salk et al. (15). HAI measures the ability of an antibody or serum sample to prevent HA binding to red blood cells, and is considered a proxy for neutralization by receptor blockade (14). HAI, however, does not fully explain or predict protection in humans (7–9). Indeed, individuals lacking detectable HAI titers were found to be resistant to influenza infection. Additionally, infection risk was clearly linked to age independently of HAI titer (7). While HAI is considered a classical surrogate of protection from influenza infection, it alone is not sufficient to fully explain protection (10).

**Figure 1:**
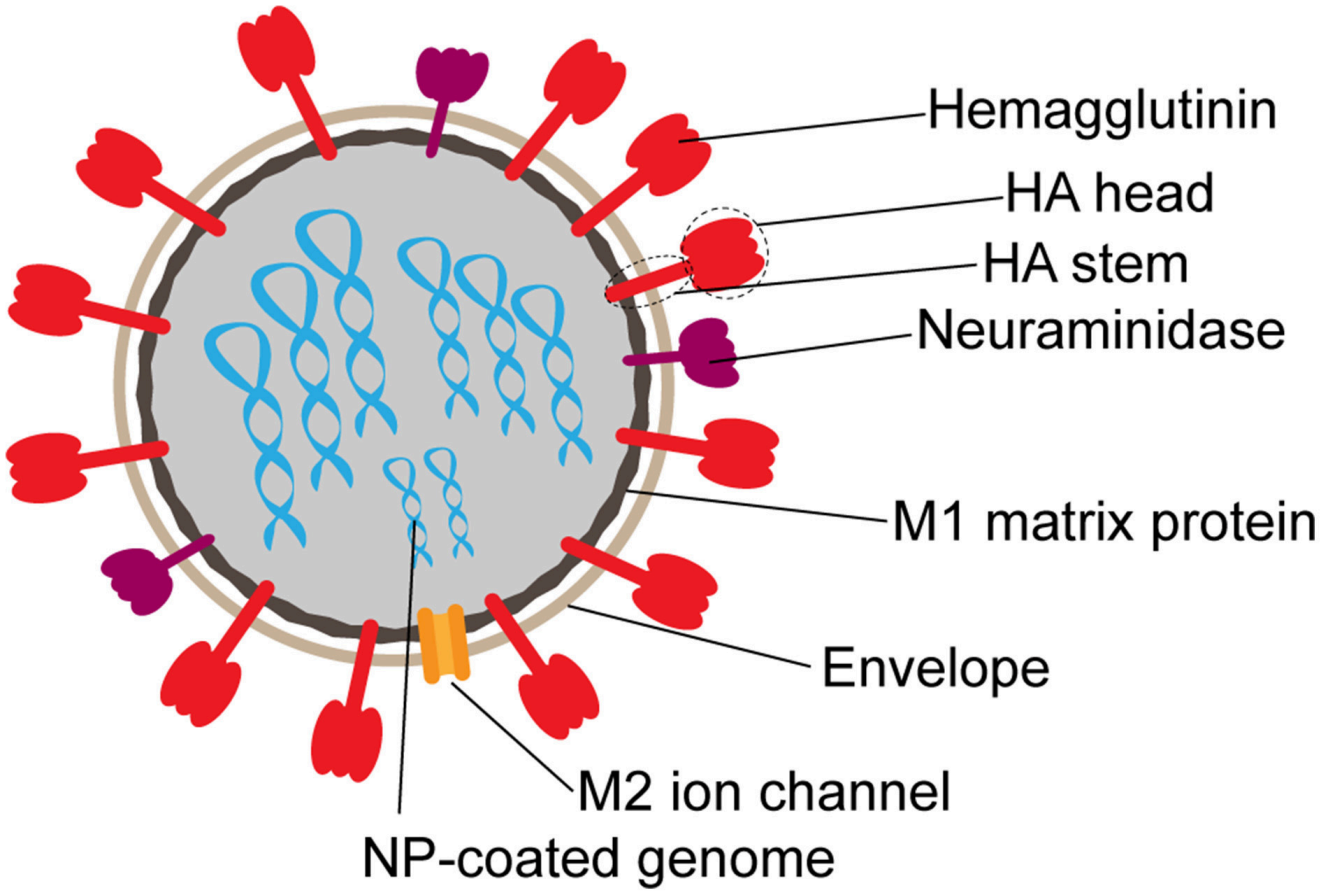
Schematic structure of influenza virion. Surface proteins hemagglutinin (HA) and neuraminidase (NA) are present on the surface at an approximate 3:1 ratio. The M2 ion channel also spans the envelope. M1 matrix protein forms the inner capsid, which surrounds the segmented RNA genome coated in nucleoprotein (NP).

Humoral immune responses to influenza are largely directed toward the hemagglutinin molecule (HA). HA, the primary viral glycoprotein, exists as a trimer made of monomers composed of two subunits, HA1, roughly corresponding to the “head” and HA2, or “stem” (16) (Figure 1). Heterosubtypic or cross-reactive, antibodies to the HA head region are relatively rare due to heavy glycosylation and low sequence conservation in this region (17, 18). Although highly variable, cross-reactive neutralizing antibodies have been discovered against the HA head. However, these antibodies bind primarily to specific conserved epitopes, including the receptor binding domain (18). In contrast, while several protective antibodies have been identified against the HA stem, as it is more conserved, this region of the HA is poorly immunogenic (17).

Heterosubtypic protective antibodies against influenza primarily target either the receptor binding site on the HA head or the more conserved HA stem (19). However, the stem region is infrequently targeted compared to easily inducible strain-specific HA head responses (20). Regardless of target, antibodies against HA can mediate protection by neutralization or extra-neutralizing functions, and both modalities may be exploited by a single antibody specificity. Antibodies that target the head largely provide protection by preventing the virus from entering the target cell, and are thus referred to as neutralizing antibodies (19). Non-neutralizing functional protective influenza-specific antibodies have been documented against both the HA head and stem (21, 22); however, their mechanisms of action are more complex and varied (23). Although protective non-neutralizing antibodies have been documented across the HA molecule, these types of protective antibodies more dominantly target the stem region of HA and can exhibit wide reactivity, capturing most influenza A viruses (20). Because the neutralizing capacity of antibodies is dose-dependent (21), in this review the term non-neutralizing will be used to describe antibodies that cannot efficiently neutralize virus at the concentration currently being studied.

**Figure 2:**
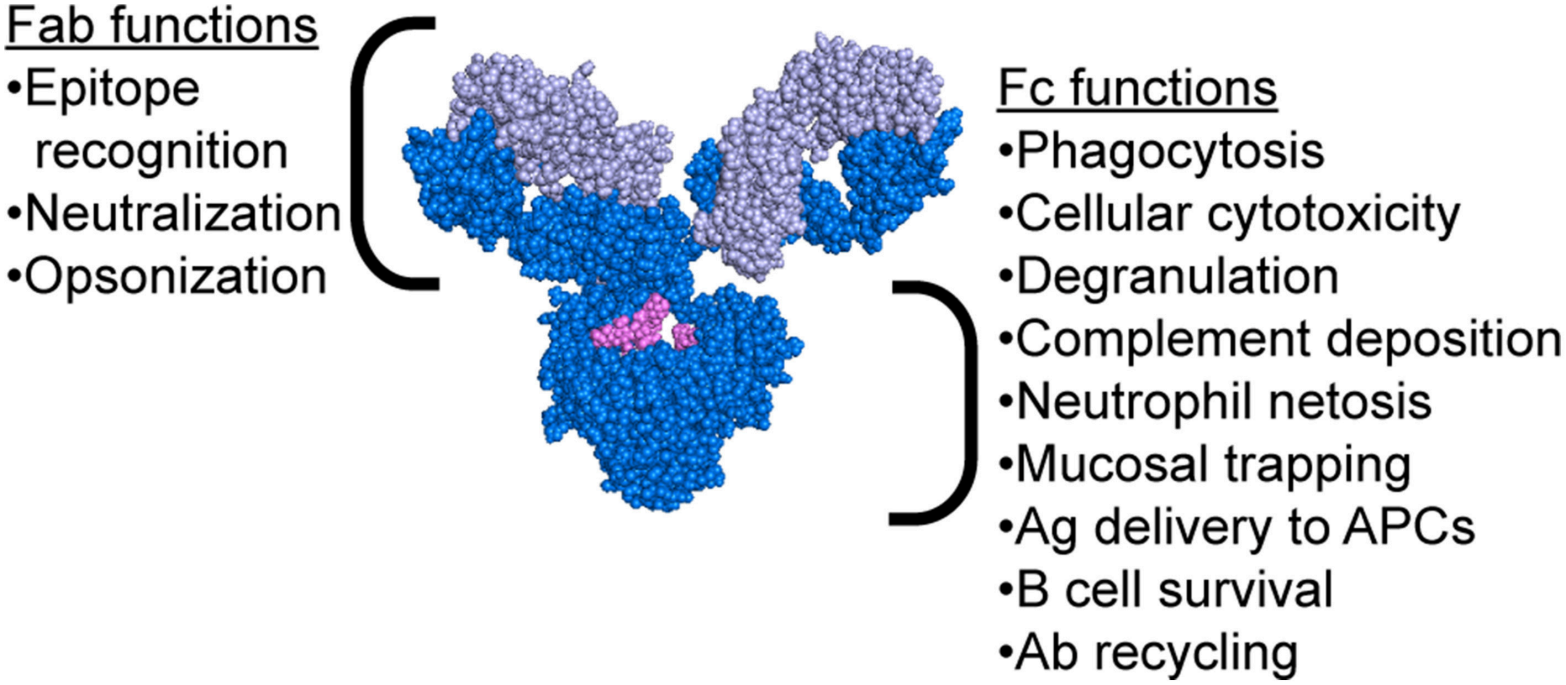
Antibody structure highlighting functions of both the Fab and Fc regions. Antibody image shows heavy chain in dark blue, light chain in light blue, and glycan in magenta. Antibody structure: PDB 1IGY.

While the mechanism of protection mediated by neutralizing antibodies is simple to comprehend, the extra-neutralizing mechanisms of action of antibodies are less well-understood. Emerging evidence has suggested that mechanisms including the ability of antibodies to leverage the innate immune system may contribute to protection against influenza (21–31). Critically, antibodies possess two functional domains: the Fab, which recognizes the antigenic epitope, and the Fc, which interacts with Fc receptors (FcR) or complement to drive antibody-mediated effector functions (Figure 2). Passive transfer studies using both native IgG1 and FcR-binding ablated monoclonal antibodies (mAbs) clearly illustrated the importance of Fc-mediated functions in protection from infection (21, 22). Moreover, follow-up studies using FcR and complement knockout mice further clarified the critical nature of specific Fc-effector functions in protection (21–23, 25–31). Antibody mediated macrophage phagocytosis (28, 32), neutrophil production of reactive oxygen species (28), cellular cytotoxicity (29), and complement deposition (26, 27, 32, 33) have all been implicated as protective functions leveraged by antibodies to drive protection from infection and/or viral clearance. Strikingly, even broadly neutralizing HA-stem targeting and pan-strain HA-head targeting mAbs require FcRs to confer protection (22). In this review, we explore the various mechanisms beyond neutralization that are exploited by antibodies to confer protection from influenza and promote viral clearance.

## FcR-mediated Functions in Influenza Infection and Vaccination

### Antibody-Dependent Cellular Cytotoxicity (ADCC) by Natural Killer (NK) Cells

**Figure 3:**
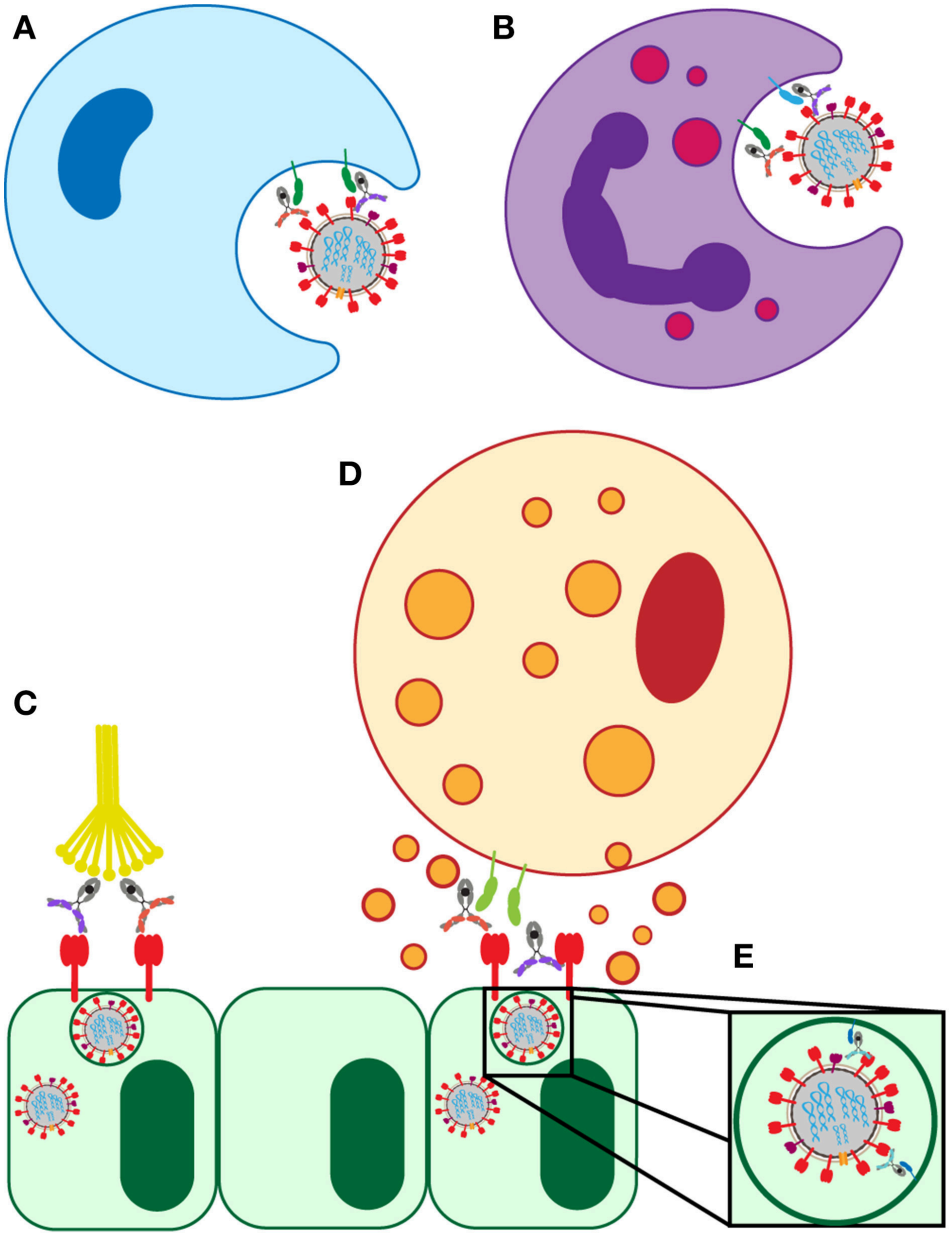
Known FcR-dependent innate immune effector functions acting in influenza infection. **(A)** Clearance of virions and infected cells by macrophage phagocytosis. **(B)** Clearance of virions and infected cells by neutrophil phagocytosis, and the release of cytokines and reactive oxygen species. **(C)** Clearance of infected lung epithelial cells and activation of the adaptive immune system by antibody interaction with C1Q. **(D)** Clearance of infected lung epithelial cells by ADCC. **(E)** Neutralization of virus by FcRn-bound HA-specific antibodies.

Antibody-dependent cellular cytotoxicity (ADCC) is largely mediated by the interaction of pathogen or cell-surface bound antibodies with Fc gamma receptor IIIa (FcγRIIIa) on NK cells in humans (Figure 3D) and FcγRIV on monocytes, macrophages, and neutrophils in mice (34, 35). FcγRIIIa is found on the surface of human NK cells, monocytes, and macrophages (36). Engagement of FcγRIIIa by antibody causes the release of cytotoxic granules from NK cells, uptake by macrophages, apoptosis of infected targets, and secretion of antiviral cytokines and chemokines (37–40).

It was first reported in 1977 that human peripheral blood leukocytes engaged in influenza-specific ADCC, and that this effect correlated with antibody-mediated virus neutralization (41, 42). *In vitro* ADCC activity was linked to protection conferred by antibodies in a mouse model of influenza infection (21, 43). Mice that received an FcR-binding competent antibody capable of inducing ADCC *in vitro* (measured by CD107a expression) exhibited increased survival and decreased morbidity compared to mice that received either an antibody unable to bind to FcRs or a less potent ADCC-inducing antibody (21). Similarly, macaque models of repeated influenza infection confirmed the rapid development of ADCC following infection in animals previously exposed to influenza. This highlighted the presence of antibodies in the bronchoalveolar lavage (BAL) capable of inducing activation of NK cells, which were associated with increased viral clearance and decreased duration of disease (44). Analogously, in human studies, ADCC titers were associated with a reduction in disease burden in a seasonal influenza experimental infection study (45). Additionally, older adults, who had previously seen 2009 pandemic influenza-like viruses in the past, and who retained long-lived ADCC, but not neutralizing, antibody titers, were protected during the 2009 H1N1 pandemic (46). This provided further evidence that ADCC-mediating antibodies are associated with protection.

However, despite our emerging appreciation for the potential role of ADCC in protection from infection and disease, the seasonal influenza vaccine poorly induced broadly reactive ADCC-inducing antibodies in healthy children and adults (45, 47–49). Conversely, the presence of cross-reactive HA-specific antibodies that can activate NK cells in older adults suggests that these functional antibodies accumulate over the course of many years of repeated natural infection with influenza (48, 50). Despite the delay in their evolution, the data clearly suggest that these functional cross-reactive antibodies emerged naturally over time. Moreover, some healthy American adults possessed ADCC activity against avian H7N9 and H5N1 viruses that do not circulate in North America but could cause pandemic outbreaks. This indicated the natural evolution of cross-reactive functional antibodies targeting diverse HA antigens in the absence of exposure and/or other conserved viral proteins such as NP and M2 (31, 51). Furthermore, broadly cross-reactive ADCC-inducing antibodies were reported in individuals who lack broadly neutralizing influenza-specific antibodies (46, 50–52), suggesting that these functions emerge separately and may evolve under distinct stimuli. Collectively, the data clearly demonstrate that broadly protective ADCC inducing antibodies are associated with protection and evolve naturally over time.

HA head-specific mAbs induced less ADCC than stalk-specific antibodies in an *in vitro* NK cell activation assay (21). This difference in function has been suggested to be related to the inability of the head-specific mAbs to efficiently multimerize when bound to antigen on the cell surface and interact with low-affinity FcRs to induce functional responses (21). A recent study suggested an alternative explanation in experiments using FLAG-tagged HA to direct FLAG-specific antibody to certain regions of the HA molecule. The data from this study suggested that two points of contact were required between infected and effector cells for efficient ADCC activity (53). These direct contacts are (1) between the mAb Fc and FcR and (2) between the cell surface sialic acid and viral HA (53). However, the co-ligation of FcR and viral protein has not been borne out by studies in other infection or disease contexts, or in polyclonal pools of antibodies directed against native HA.

An additional layer of complexity in dissecting non-neutralizing antibody mediated mechanisms of protection *in vivo* is the comparison of polyclonal vs. monoclonal mediated antibody functions. Emerging data suggest that the level of *in vitro* ADCC is influenced by the ratio of ADCC-inducing to ADCC-inhibiting antibodies (22, 54). ADCC-inhibiting antibodies, which can be neutralizing, were shown to compete for binding sites on HA on the surface of viral particles and infected cells (22, 54). While the delivery of single protective ADCC-inducing mAbs demonstrated striking protection from infection *in vivo* (21, 22), polyclonal pools of antibodies exhibited a much more complex balance of epitopes targeted and functional competition that collectively may contribute to differential protection from infection during seasonal exposure. While it is clear that functional antibodies play a vital role in protection against influenza infection, experimental approaches able to comprehensively dissect the nature of polyclonal antibody interactions are urgently needed to further define the nature of protective antibody activity and guide vaccine design.

### Antibody-Dependent Macrophage Phagocytosis and Activation

ADCC-inducing antibodies, as well as the direct cytopathic effects of the virus, drive infected cell apoptosis (55). These infected apoptosing cells are then cleared through phagocytosis to maintain tissue homeostasis (56). Post-infection, macrophages are rapidly recruited to the lung and are present in BAL, airway, and alveoli to support the rapid clearance of infected and/or dying cells (57). While the supernatant of influenza-infected cells can stimulate monocyte phagocytosis independently of antibody involvement (57), antibodies contribute to accelerated clearance of viral particles and infected cells through interactions with FcγRIa and FcγRIIa on immune cells (58). Antibody mediated viral phagocytosis, resulting in viral degradation, was linked to decreased spread and severity of infection (58). While this mechanism was not directly associated with prevention of infection, it was linked to reduced severity of symptoms and viral shedding, and thus attenuating disease in humans.

Antibody-dependent cellular phagocytosis (ADCP) activity (Figure 3A) in healthy human serum, mediated by monocytes/macrophages, was shown to correlate with HAI titer both for circulating and non-circulating strains of influenza (58). Interestingly, ADCP activity was still detectable in diluted serum samples, even at dilutions where neutralization was no long detectable (58). This indicated that phagocytic antibodies may mediate viral clearance even at very low levels, and thus could still provide protection or lessen the severity of disease. Along these lines, non-neutralizing protective mAbs in mice required alveolar macrophages to provide protection. This protection was partially dependent on the induction of a robust inflammatory response in the lung as shown by tissue histology and increased cytokine/chemokine production, and was partially through direct phagocytosis (30). Additionally, broadly neutralizing HA-specific mAbs also exhibited enhanced protection in the presence of alveolar macrophages (30). This macrophage-mediated protection was dependent on interactions of the antibody with FcRs on the macrophage surface, as evidenced by experiments using FcR-binding null antibodies that failed to provide protection from infection (30). Together, these studies indicate that FcR-mediated macrophage activation reduces disease burden and protects mice from lethal influenza, and that healthy human serum has influenza-specific antibodies capable of inducing this function.

### Antibody-Dependent Neutrophil Phagocytosis and Activation

Neutrophils are among the first cell populations recruited to the site of infection and/or inflammation, and have been implicated in the protective response to influenza (59). Neutrophils are involved in the phagocytic clearance of both virions and infected cells, release immunostimulatory cytokines and chemokines to recruit additional immune cells, and form neutrophil extracellular traps (NETs) to capture and inactivate the virus (60). During influenza infection, neutrophils generate the chemokine CXCL12, required for efficient recruitment of cytotoxic CD8^+^ T cells to the lung (61). However, beyond this indirect anti-viral role, human neutrophils express high levels of FcγRIa/b/c, FcγRIIa, and FcγRIIIb after activation, enabling them to respond rapidly and efficiently to antibody coated targets (36). In addition, neutrophils constitutively express the FcαRI, an activating receptor that binds IgA and activates cytotoxic and phagocytic responses via a shared FcR γ-chain (62).

Like macrophages, neutrophils are intimately involved in the phagocytic clearance of infected and apoptotic cells in the lung during influenza infection (Figure 3B) (57). Impaired neutrophil phagocytosis through depletion of neutrophils was linked to decreased survival in a mouse model of influenza infection (57, 63). Following phagocytosis, neutrophils form phagolysosomes containing reactive oxygen species (ROS) to eliminate the virus (60). Both HA head- and stalk-specific mAbs induced the production of ROS by neutrophils *in vitro* in an FcR dependent manner, shown following Fc-blockade resulting in reduced ROS production (28). Influenza-specific class-switched IgA antibodies were also implicated in neutrophil activation and ROS production (28). Despite the ability of some antibodies to recruit neutrophil activity *in vitro*, the critical nature of neutrophils in protection from infection remains controversial. Some animal studies using neutrophil depletions finding no significant roles for these cells in protection mediated by passively transferred non-neutralizing mAbs (30), arguing that alveolar macrophages may play a more dominant role in protection. In other studies, neutrophil recruitment and function was linked to protection from infection and reduction in disease (63, 64). Thus, additional studies will be required to ultimately define the role of neutrophils in influenza infection.

### Antibody-Dependent Complement Activation

The complement system can recognize and eliminate viruses directly or can contribute to viral clearance via antibody mediated activation (Figure 3C) (33). The requirement for complement in protection from lethal influenza infection in mice was established in 1978 and has been more recently replicated on novel influenza strains (26, 65). Influenza virions were shown to be susceptible to both classical and alternative complement mediated lysis *in vitro* only when opsonized by antibodies (27). However, the level of susceptibility varied by strain. Further supporting the involvement of antibody-mediated complement elimination in the influenza immune response, synergy between the classical and alternative complement pathways was shown to provide protection against pandemic H1N1 strains in mice and the cooperativity of both pathways is associated with enhanced viral clearance (27). In these experiments, C3 knockout mice (deficient in all complement pathways because C3 is the central point of the cascade), C4 knockout mice (deficient in classical and lectin pathways), and complement factor B (FB) knockout mice (deficient in alternative pathway) were infected with influenza and disease progression was compared. While both C4- and FB-deficient mice showed increased mortality, neither pathway alone nor the additive mortality approached the level of mortality in C3 knockout mice, who have both pathways of complement ablated. This indicated that the two complement pathways work synergistically to clear infection (27).

Beyond antibody driven virion elimination, the complement protein C3 was also shown to promote higher titers of influenza-specific IgG antibodies. C3 also improved CD4+ and CD8+ T cell responses in the mouse models of influenza infection (33). Vaccination was administered to C3 knockout mice, resulting in dampened antibody titers leading to increased mortality when compared to wild type mice. The role of complement in driving immunity was proposed to be effectuated by the formation of pro-inflammatory complement degradation products C5a and C3a, which can serve a dual role of directly recruiting T cells and enhancing T cell priming by recruiting and stimulating antigen-presenting cells to the site of infection (33).

In human serum, neutralization and complement-dependent lysis activities by mAbs have not always correlated, although neutralizing antibodies can induce complement-dependent lysis (66). Both IgG1 and IgM antibodies have been implicated in the activation of the complement system in influenza infection (27). Complement-stimulating antibodies correlated with protection from infection in children in a serosurveillance study of seasonal influenza (50), which was potentially attributable to their generally higher cross-reactivity compared to neutralizing antibodies (31). Importantly, if complement-inducing antibodies do in fact generally possess higher cross-reactivity when compared to neutralizing antibodies, complement lysis of virus is an attractive strategy for limiting initial infection with influenza by otherwise non-protective non-neutralizing antibodies, broadening the epitopes that can be targeted by vaccination.

### Additional Functions Via Non-classical FcR

The neonatal Fc receptor, FcRn, is involved in transcytosing IgG across the placenta during fetal development, across the vascular endothelium to increase extravascular antibody levels, and across the mucosal epithelium to provide humoral defense within the mucosa (67). Additionally, FcRn has a non-canonical role in antiviral immunity against influenza. FcRn was implicated in facilitating antibody-mediated neutralization of influenza virions by HA head-specific antibodies that bind to the virus at acid pH (Figure 3E) (68). These unusual head-specific antibodies were then shown to neutralize the virus by preventing trafficking of the viral ribonucleoproteins into the nucleus for replication (68).

Systems level analyses aimed at defining biomarkers of productive immunity to flu vaccination identified pre-existing antibody titers as a negative predictor of response to vaccination (69), thought to act by capturing, destroying, and preventing response to vaccine antigens (historically called original antigenic sin) (70). However, recent studies suggested that pre-existing antibodies shape the immune response to influenza vaccination in ways that could be utilized to improve protection. Individuals with the most influenza-specific antibody affinity maturation had significant changes in antibody glycosylation, namely increased sialic acid (71). A mechanism was proposed in which pre-existing cross-reactive influenza antibodies, which opsonize incoming vaccine antigen, drove the delivery of immune complexes to germinal centers of lymph nodes by subcapsular sinus macrophages or non-cognate B cells, preferentially when antibodies were sialylated (72, 73). This delivery relied on interaction of immune complexes with the non-canonical IgG Fc-receptor CD23 to capture antigen and move it to germinal center (71). This delivery of antigen to lymph nodes was speculated to increase and extend the contact between B cells and antigens of interest to drive affinity maturation, which can increase both the affinity and potential evolution of neutralization (74, 75). Identified broadly cross-reactive neutralizing mAbs specific to influenza HA were shown to be highly affinity matured, indicating that this pathway may be essential for the development of broad humoral immunity (76).

In addition to trapping and delivery, these sialylated Fcs were also shown to increase B cell inhibitory FcR, FcγRIIb, expression, resulting in elevated thresholds required to activate B cells during development in the germinal center (77). With these elevated activation thresholds, B cells that require higher affinity interactions or an ability to capture more antigen to become fully activated within the germinal center may then experience more aggressive somatic hypermutation and consequent affinity maturation. Sialylated immune complexes bind to non-cognate B cells at high levels in the presence of CD23 (77) to increase affinity maturation. This may offer a novel approach for the design of next generation vaccines able to leverage the potent immunomodulatory activity of the Fc-domain of antibodies.

Together, these data suggest that the quality of the antibody response may not only influence direct antiviral activity, but may also be critical in influencing the response to vaccination. Seasonal influenza infection in humans has been shown to induce moderate antibody cross-reactivity (78), and these pre-existing cross-reactive antibodies may be molding the affinity maturation of new antibodies following vaccination through mechanisms that increase somatic hypermutation, including increased antigen retention within germinal centers (71). Harnessing, increasing, and improving this pathway presents a novel method of improving the breadth and binding affinity of antibodies following influenza vaccination.

## Control of FcR-mediated Functions by Antibody Properties

### Subclass and Isotype Variation

**Figure 4:**
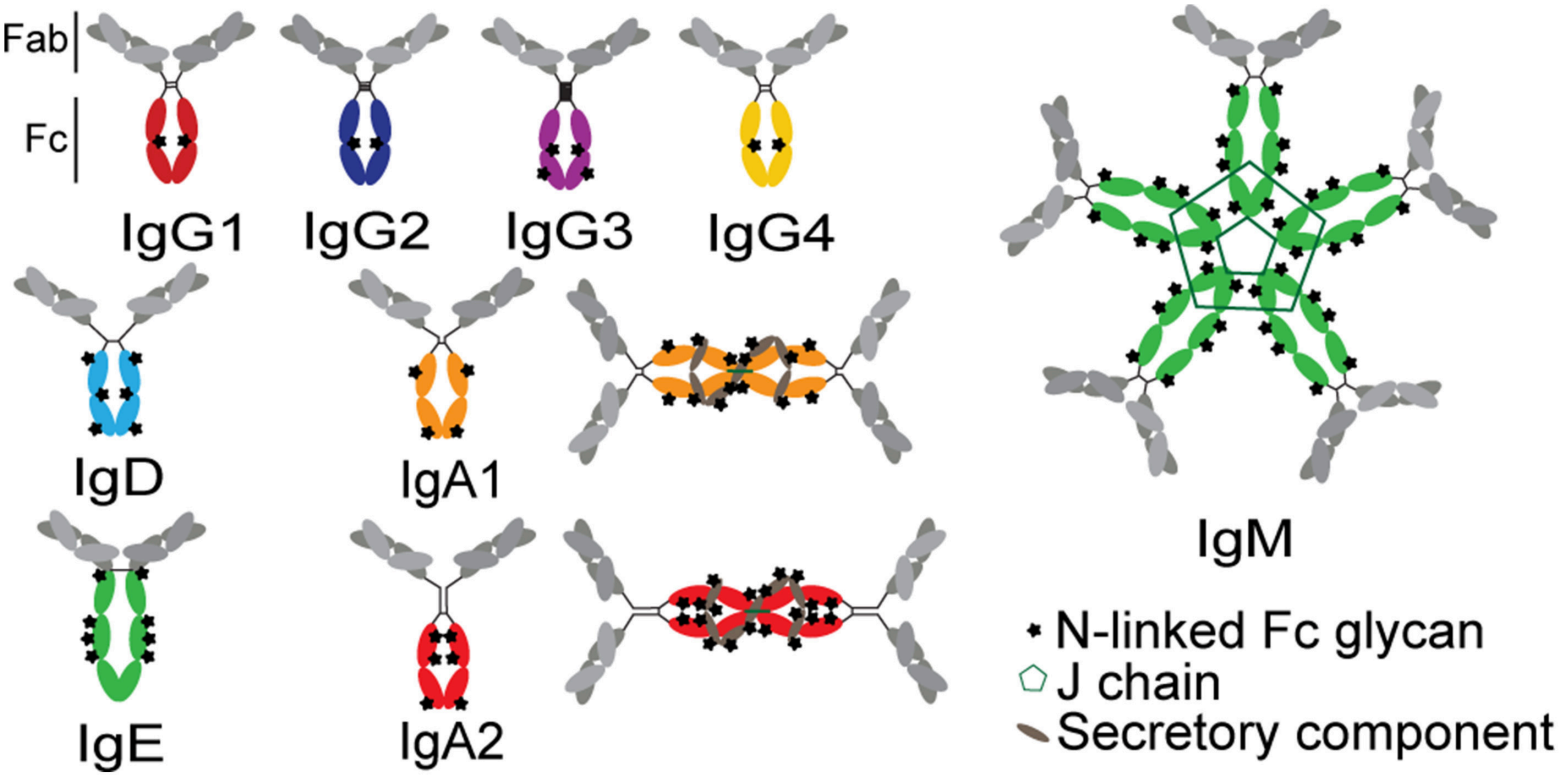
Structures of antibody isotypes and subclasses. Fc domains are in color while Fab domains are in gray. Stars indicate N-linked Fc glycans. IgA isotypes are shown as both monomers (predominant in serum) and dimers (predominant at mucosal surfaces).

The functional potency of an antibody is significantly affected by the antibody's subclass, which determines the binding affinity of the antibody for FcRs (79). Antibody function is determined at the time of B cell programming via class switch recombination (IgA, IgM, IgG, IgD, or IgE; Figure 4). Because each isotype can interact with a distinct family of FcRs present on innate immune cells within disparate compartments, each isotype has the capacity to drive unique antibody effector functions. Beyond the isotypes, there is additional capacity to select for subclasses of particular antibody isotypes. In humans, four IgG subclasses can be additionally selected during an immune response, each of which have further differential affinities for individual FcRs (36). IgG1 antibodies are the most prevalent at approximately 65% of total serum IgG, with the other three subclasses in decreasing fractions in numerical order (80). Because individual subclasses have different affinities for FcRs (36), subclasses drive different antibody effector functions. IgG1 and IgG3 are considered to be the most functional subclasses due to their enhanced ability to bind to FcRs, while IgG2 and IgG4 have lower affinities for FcRs (81, 82). However, IgG3 has a shorter half-life, related to decreased binding affinity to FcRn and due to a proteolytically vulnerable hinge, although multiple allotypes of IgG3 with longer half-lives have been reported among non-Caucasian populations (83).

The relative magnitude and distribution of IgG subclass responses vary between acute influenza infection and vaccination. Vaccination increased IgG3 production when compared to acute infection in adults and children who were previously exposed to natural influenza but not previously vaccinated (84). IgG3 levels following seasonal influenza vaccination correlated with cytokine production by peripheral blood mononuclear cells (PBMCs) stimulated *ex vivo* with infectious influenza virus, suggesting that enhanced IgG3 responses were a marker of a more effective response to vaccination (80). While IgG3 is widely considered to be the most functional subclass due to its affinity for FcRs, the specific effects of IgG3 in protection from influenza remain largely unclear.

While IgG is present at higher levels in the blood, IgA antibodies are produced at considerable levels in mucosal tissues (85). Secreted IgA represents ~70% of the body's total Ig production and, in mucosa, is primarily dimeric, with only small fractions of monomer, trimer, and tetrameric IgA in the mucosa. In serum, IgA is primarily monomeric (86). Mucosal IgA can prevent influenza infection in the nasal and upper respiratory mucosa with higher heterologous neutralization than IgG with the same Fab (85, 87–89). The protective activity of IgAs was linked to both direct viral neutralization as well as viral capture and cross-linking to the mucosal surface, preventing cell entry in the absence of classical neutralization (85). However, beyond its direct antiviral effects, IgA may also recruit the indirect activity of the innate immune system, via the Fc-receptor for IgA, FcαRI, which is constitutively expressed on neutrophils and increases in expression as neutrophils mature (62). Stimulation of this receptor by influenza-specific IgA was linked to increased ROS production (28), although the precise effect of this activation during influenza infection is unclear.

### Antibody Fc Glycosylation

**Figure 5:**
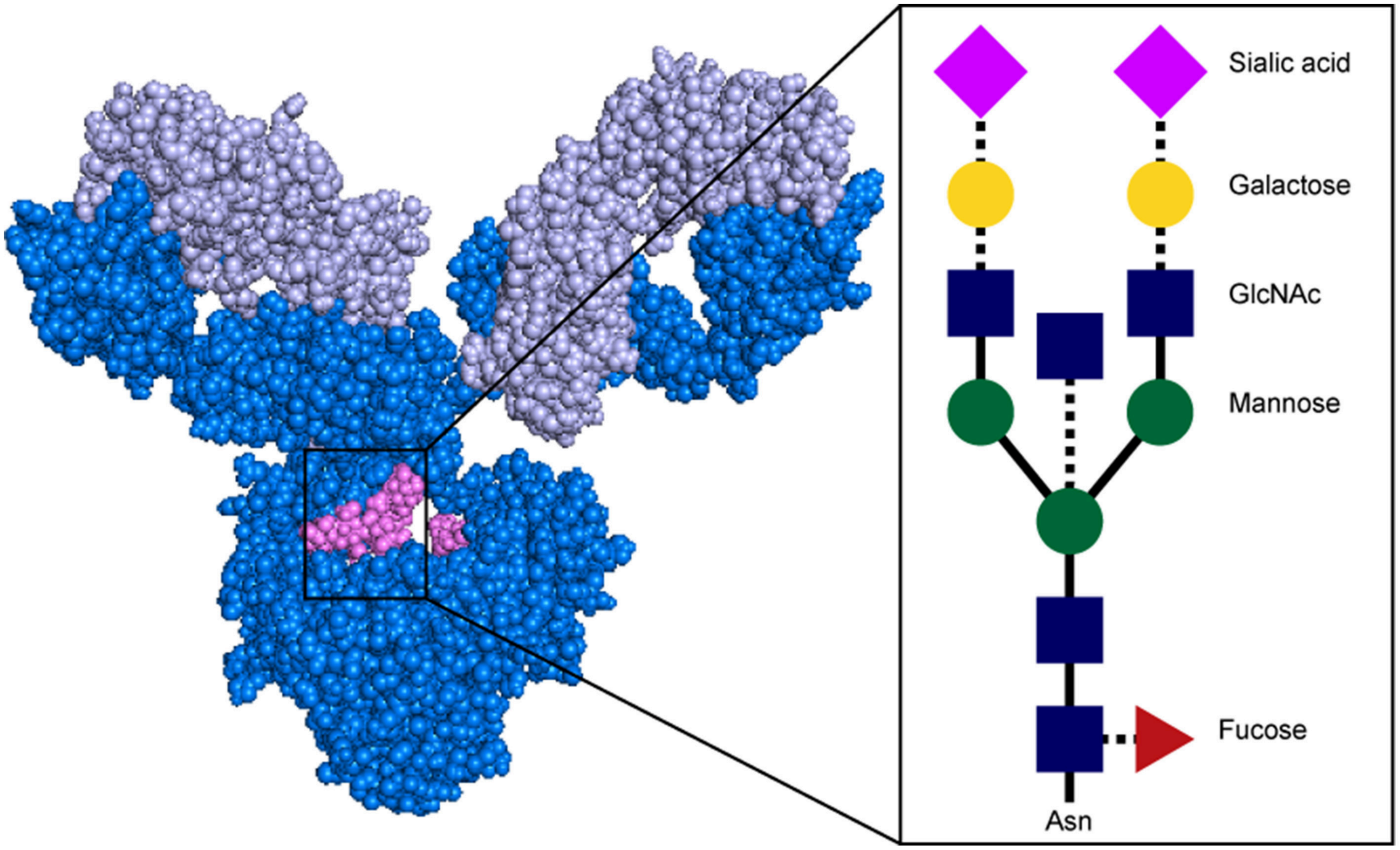
Structure of antibody glycan. Antibody image shows heavy chain in dark blue, light chain in light blue, and glycan in magenta. In glycan schematic, solid lines indicate core glycan consisting of two-branched linked N-acetylglucosamine (GlcNAc; blue rectangle), a mannose (green circle), followed by 2 branched mannoses, each followed by an additional GlcNAc on each mannose. Dotted lines indicate additional sugars that can be added at variable levels, including a core fucose on the first GlcNAc (red triangle), galactoses (yellow circle) to each terminal GlcNAc, sialic acids (pink diamond) to the each galactose, and a bisecting GlcNAc to the core mannose. Antibody structure: PDB 1IGY.

Beyond isotype and subclass selection, the humoral immune response additionally modifies antibodies via post-translational changes in Fc-glycosylation to further tune antibody affinity for FcRs, and thus to modulate antibody effector function (90). Each IgG molecule contains two N-glycosylation sites, at asparagine 297 (N297) on each heavy chain (Figure 4). The core Fc glycan structure is biantennary, with a structure consisting of two-branched linked N-acetylglucosamine (GlcNAc), a mannose, followed by 2 branched mannoses, each followed by an additional GlcNAc on each mannose (Figure 5). Three additional sugars can then be added at variable levels, including a core fucose on the first GlcNAc, galactoses that can be added to each terminal GlcNAc, sialic acids that can subsequently be added to the each galactose, and finally the addition of a bisecting GlcNAc to the core mannose (Figure 5) (91). Given the variable addition of each of the 4 additional sugars, a total of 36 distinct glycan structures can be added to any given IgG (92). Importantly, while glycans do not interact directly with FcRs, they influence the flexibility and structure of the antibody Fc, thereby changing interactions with FcRs (93). Complete removal of the Fc glycan ablates low affinity FcR binding, with only high affinity FcγRI retaining measurable binding ability (94). Additionally, IgA and IgM antibodies are also Fc-glycosylated (Figure 4), although it is unclear how this glycosylation changes affinity for FcRs.

Across diseases, dramatic shifts have been identified in IgG glycosylation, such as a significant increase in agalactosylated antibodies in chronic inflammatory diseases such as autoimmune flares and HIV infection (95, 96). In the monoclonal therapeutics community, the systematic removal of specific components of the Fc N-glycan have highlighted the critical role of individual sugars in shaping antibody effector function (97). Specifically, the presence of sialylation drives anti-inflammatory activity *in vivo* (98, 99). The removal of fucose either directly or indirectly, through the upregulation of the bisecting GlcNAc, results in increased antibody affinity for FcγRIIIa and consequently enhanced ADCC (90, 100). Conversely, agalactosylated antibodies decrease ADCC and drive pro-inflammatory responses (90).

Beyond our emerging appreciation for a role of sialylated antibodies in vaccine induced affinity maturation (71, 101) described above, influenza vaccination is known to alter Fc glycosylation (102, 103). Soon after immunization, influenza-specific antibodies rose rapidly, and had increased galactosylation, increased sialylation, and reduced bisection compared to pre-existing influenza-specific antibodies (102, 103). However, these glycan shifts normalized after a month, suggesting that these transient changes in influenza-specific antibody glycan profiles may reflect differences in glycosylation by plasmablasts, not plasma cells. Even in the absence of vaccination, HA-specific antibodies exhibited unique glycan profiles compared to HIV-specific antibodies from the same individuals. Specifically, HA-specific antibodies were more highly galactosylated and sialylated and contained reduced b-GlcNAc (102), highlighting the unique glycan profiles that are naturally selected on influenza-specific antibodies. Whether individuals who control the virus more effectively tune antibody glycosylation in a specific or selective manner is unclear, but represents a simple strategy to modulate antibody function.

## Optimizing Antibody Responses Through Vaccination

### Adjuvants

One of the greatest hurdles of influenza vaccination is overcoming response anergy caused by previous exposures to influenza in the aging immune system. An attractive strategy to overcome this anergy and generate protective humoral immunity, particularly for novel strains or universal vaccine formulations, is the use of adjuvants to enhance and tune the response to vaccination. There are currently four adjuvants licensed for use in influenza vaccines in the United States and/or in Europe: aluminum salts (alum), MF59, AS03, and virosomes (104). In addition to adsorbing antigens and creating multivalent lattices of antigens, alum activates the inflammasome promoting more effective responses upon antigen-presenting cell delivery (105). Alum induces primarily a Th2-driven response (104). However, Th1 responses are likely to be more critical in the clearance of intracellular pathogens, including influenza (105). Oil-in-water emulsions, such as MF59 and AS03, are predicted to work through a more balanced Th1/Th2 response, enhancing both T cell and antibody responses via delivery to antigen presenting cells as well as through the recruitment of specific innate immune cells to the site of injection (106). Specifically, in an adjuvanted HIV vaccine trial in macaques, MF59 increased recruitment of neutrophils, monocytes, and MDCs to the site of injection and recruitment of neutrophils to the draining lymph node (107). In the context of influenza vaccination, MF59 increased the heterosubtypic, or broadly reactive, antibody response and increased neutralizing antibody responses to influenza (108, 109). Unfortunately, MF59 also shifted the response even further toward the immunodominant HA head and away from the HA stem (110). Yet, MF59 increased the affinity of antibodies developed following both seasonal and novel pandemic influenza vaccines, suggesting that if skewed selectively to particular target antigenic sites, this adjuvant could drive enhanced affinity maturation to the correct sites of vulnerability (110). Virosomes or phospholipid vesicles, have also been studied in the context of influenza HA and NA vaccines, showing similar profiles to MF59 (104). The effects of these adjuvants on FcR-mediated antibody functionality are only beginning to be studied (107).

Other adjuvants are currently under investigation to specifically and selectively enhance influenza specific immunity. For example, liposomes provide unique scaffolds for antigen delivery (105), and were shown to increase the humoral and Th1 response, boosting neutralization, in mice following influenza vaccination (104). Additionally, virus-like particles, or nanoparticles, which deliver antigens in a multivalent manner, similar to their native conformation, increased heterosubtypic IgG2a neutralizing antibody titers in mice, the mouse analog of IgG3, the most functional antibody subclass in humans (111). Presentation of antigens in the form of a viral particle may play an essential role in driving functional antibody responses (112–114). Another type of adjuvant, ISCOMS (antigen, cholesterol, phospholipid and saponin-defined immunomodulatory complexes), created a balanced, protective immune response based on strong MHC class I presentation in trials with a pandemic influenza antigen (104). However, tests of ISCOMS with cancer antigens showed that this adjuvant shifted the response away from antibodies, toward CD4+ and CD8+ T cells, with limited changes seen to antibody responses (115). Finally, Toll-like receptor (TLR) agonists, involved in early pathogen sensing, are known to tune the inflammatory response to tailor immunity in a pathogen specific manner (104). Several TLR agonists were shown to increase influenza-specific antibody titers following vaccination, however their effects on antibody-mediated functions beyond neutralization are unexplored (104, 116). Thus, while previous studies with these adjuvants have primarily focused on neutralizing antibody responses, additional insights on the specific effects of adjuvants on shaping protective FcR activity will provide additional avenues to tune and direct protective immunity against influenza infection.

### Antigen Design and Glycosylation

In addition to efforts to promote more effective immune stimulation through adjuvants, intense investigation has focused on the development and design of unique antigens able to selectively direct the immune response away from strain-specific immunodominant sites to those that are more conserved (17). These include the design of computationally enhanced globally relevant HA sequences, the design of mini-antigens and chimeric antigens, and glycan-enhanced antigens.

Computationally optimized broadly reactive antigens (COBRAs), designed based on computational modeling of influenza strains to create mosaic antigens aimed at focusing the immune response on the evolution of heterosubtypic responses, had some success in eliciting broadly reactive HAI titers that target both seasonal and pandemic strains of influenza (117–120). In a recent study, COBRA H3s did not increase the breadth of HAI reactivity by vaccine-induced antibodies across a panel of strains. However, these COBRA H3s did increase the phylogenetic diversity of neutralized strains (120), meaning that the COBRA-induced responses altered which strains were recognized and neutralized without increasing the total number of strains recognized (120). These data suggest that COBRA antigens can increase heterosubtypic responses to conserved epitopes on the immunodominant head, but are unable to create broadly reactive responses to the conserved HA stem.

Given the complexity of altering immunodominance using whole HA molecules, additional efforts have aimed to direct immunity against minimal antigenic regions associated with broadly protective responses including the stem (121, 122) and the receptor binding site (123). Although broadly protective non-neutralizing responses can target the stem region of HA, these responses are typically subdominant (124). HA stem-only antigens or antigens with conserved stem domains but altered HA heads have been developed (17). For example, a “headless” HA vaccine tested in mice created a broadly protective non-neutralizing immune response (125). Furthermore, chimeric HA vaccines, which were used to immunize animals with “exotic” chimeric molecules that coupled unusual heads to a single stem region, have shown promise. Specifically, using heads that have not circulated in the population, coupled to conserved stems, this vaccine strategy drove robust focused stem-specific protective immune responses and higher cross-reactive HA-specific antibody titers, and are now in clinical trials (126, 127).

Seasonal influenza vaccines have been produced in eggs since the introduction of yearly vaccination, and the manufacturing techniques have remained largely unchanged for decades (128). Emerging data and technical advances are increasing the attractiveness of cell culture-based production strategies, rather than egg-based production. Beyond issues related to speed and cost of vaccine production across these platforms, qualitative differences in antigens from egg-based vaccines compared to circulating viruses may necessitate this shift. HA is highly N-glycosylated in a host-cell dependent manner (129–131). The glycosylation of egg-grown vaccine virus is different than that of naturally infecting virus (132). Emerging data points to the importance of glycosylation not only in shaping antigen-exposure on the surface of the HA molecule, such as masking of specific epitopes (131, 133), but also in contributing to the antigenicity of mAb binding epitopes (134–136). Differential HA glycosylation between egg- and cell culture-grown virus impacted innate immune interactions with the virus in the lung, including neutralization by surfactant protein D (SP-D) (137) and binding to mannose-specific lectins (138). Moreover, altered glycosylation was shown to change both cellular and humoral response kinetics *in vitro* and *in vivo* (131, 139). Vaccination of mice with de-glycosylated HA led to decreased CD4+ T cell activation and cytokine production, resulting in reduced HA-specific antibody titers and HAI titers (131, 139). Studies of the antibody response using differentially glycosylated (not de-glycosylated) HAs showed that glycosylation alters the binding and neutralization of monoclonal antibodies, but lacked further detail about the effects of glycosylation on polyclonal antibody pools or on Fc-mediated function (131, 139, 140).

Epidemiological studies in recent years investigating poor vaccine protective efficacy have shown that antigen glycosylation had a direct impact. In the 2016–2017 flu season, the circulating H3N2 virus had a new glycosylation site compared to previous seasons. However, the egg-adapted version of the viral strain used to produce the vaccine lacked this site through an amino acid mismatch in an antigenic site, resulting in decreased vaccine effectiveness (134). Given that glycosylation can strongly impact epitope antigenicity, a vital mismatch at a site of neutralization sensitivity resulted in the induction of non-protective immunity and rendered the circulating virus invisible to vaccine induced immune responses. Shaping glycosylation to produce representative antigens is critical to achieving protective immune responses to vaccination.

### Additional Antigenic Targets

While the majority of the humoral immune response is directed toward the immunodominant HA molecule, antibodies also emerge against other targets including neuraminidase (NA), nucleoprotein (NP), and Matrix-2 (M2) (Figure 1). Antibodies targeting NA, while not neutralizing, can prevent viral exit from infected cells to block subsequent rounds of infection (12), and have been associated with seasonal protection (12, 141–143). However, NA-specific antibodies have also been shown to drive ADCC (51, 144), suggesting that this less immunodominant target is vulnerable to multiple modes of antibody mediated targeting.

The highly conserved internal viral proteins NP and M2 have been shown to induce an immune response that is also broadly reactive (145). NP-specific antibodies, which are always non-neutralizing (146), mediated viral clearance through FcRs and protection in mouse models of influenza infection (143, 147). Similarly, non-neutralizing M2-specific antibodies mediated ADCC and ADCP to clear infected cells and promoted rapid viral clearance (141, 142). Thus, while current vaccination strategies largely focus on the development of broadly reactive immunity against HA, additional largely non-neutralizing conserved antigens exist within influenza that may represent next generation targets for protective universal immunity.

## Conclusion

More than 4 decades of research has clearly illustrated the importance of both direct neutralization and non-neutralizing functional antibodies in protection against influenza infection and disease. Because neutralizing and non-neutralizing antibody activities are not induced in a mutually exclusive manner, vaccine strategies able to leverage both functions of antibodies are likely to confer the greatest level of protection. However, the precise innate immune effector functions to precise sites of viral vulnerability on HA or other target antigens remain to be determined. With emerging novel vaccine design strategies, coupled to emerging immune modulatory adjuvants, opportunities to drive universal protection are on the horizon.

## Author Contributions

CB and GA wrote the review article, contributed to revision, and approved the submitted version.

### Conflict of Interest Statement

The authors declare that the research was conducted in the absence of any commercial or financial relationships that could be construed as a potential conflict of interest.
